# Supplementary figures and images for: Pharmacological and non-pharmacological methods of inducing wakefulness activate distinct neural populations in the mouse brain
Source: PLoS Biol. 2026 Mar 19;24(3):e3003622. doi: 10.1371/journal.pbio.3003622 (PMC13038112; doi:10.1371/journal.pbio.3003622)

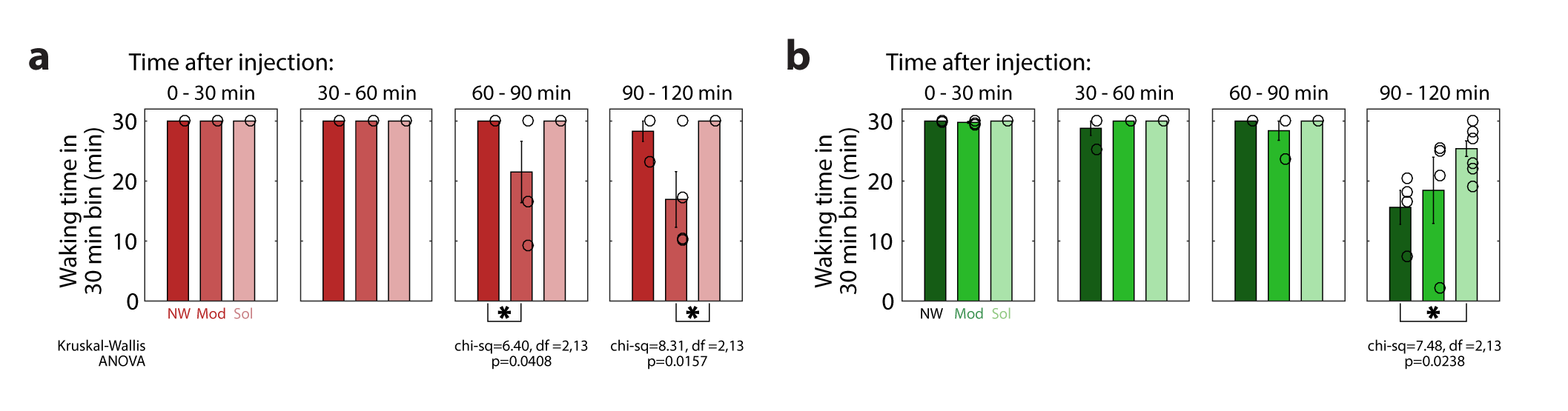

Supplement: S1 Fig — Time spent in wakefulness in the first (a) and second (b) experimental session per 30 min, starting at the injection of solriamfetol (Sol), modafinil (Mod) or the beginning of the induction of NW, open field). Nonparametric Kruskal-Wallis followed by post hoc Mann-Whitney test, * p < 0.05. (TIF) [file pbio.3003622.s001.tif]

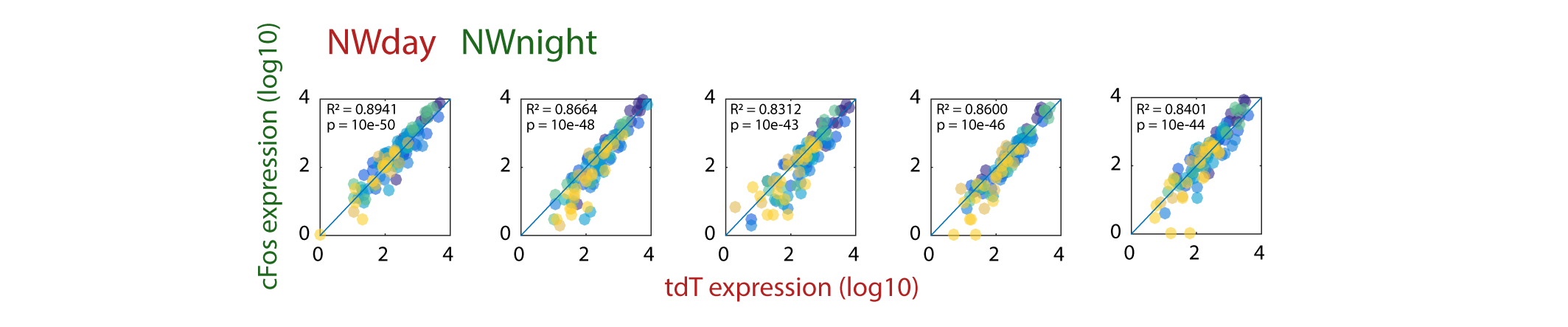

Supplement: S2 Fig — Scatter plots show log10-transformed tdT expression versus log10-transformed cFos expression for individual mice submitted to the circadian protocol. As shown for the previous groups, Nwday (tdT expression) and Nwnight (cFos expression) were highly correlated. Each dot represents one structure of a given macrostructure (color coded). The diagonal indicates the linear regression fit. Coefficients of determination (R2) and corresponding p-values are shown in each panel, demonstrating a strong and highly significant positive correlation between tdT and cFos expression across all mice. Raw data underlying the Figure is shown in S3 Data. (TIF) [file pbio.3003622.s002.tif]

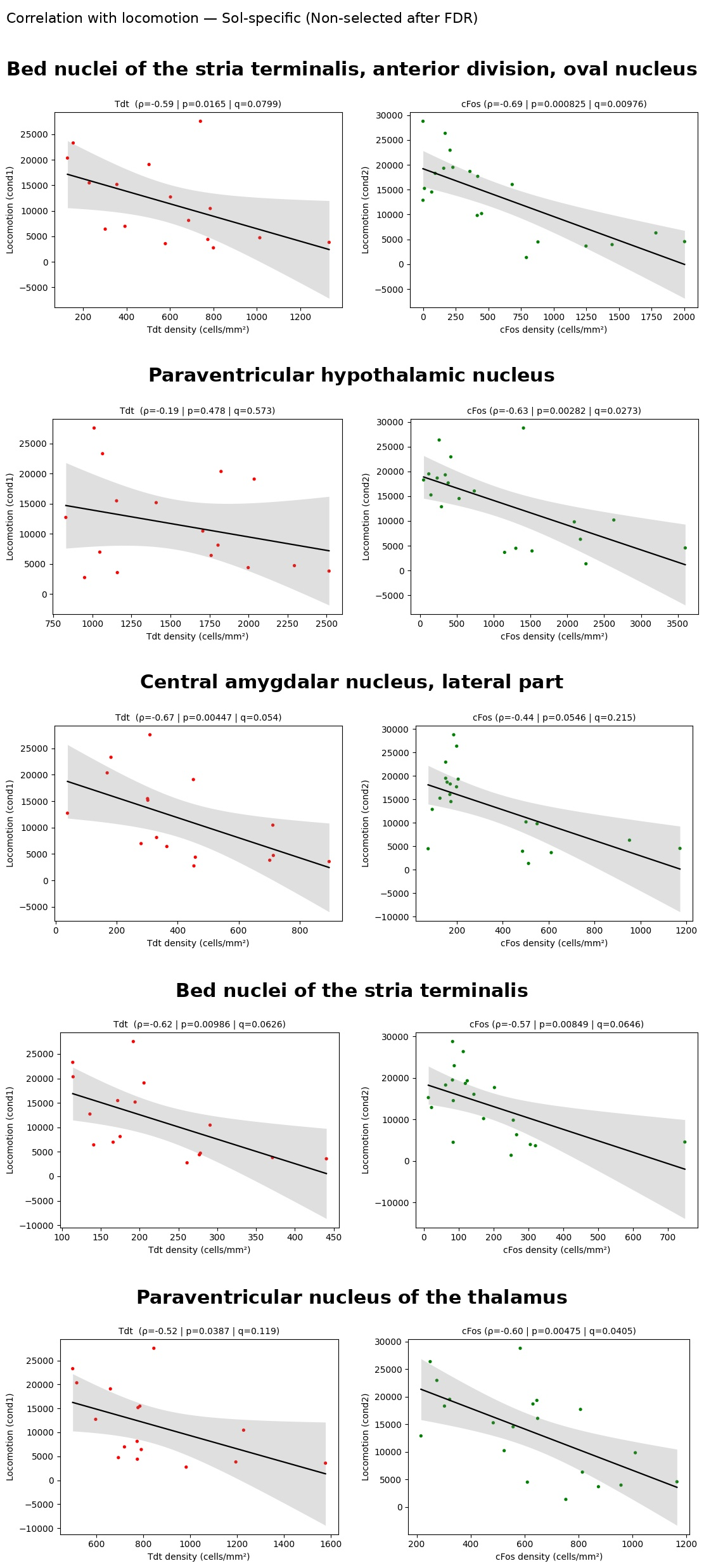

Supplement: S3 Fig — Panels illustrating the five Sol-specific regions that did not show a significant correlation between locomotion and neuronal density across both markers after Benjamini–Hochberg FDR (q < 0.05) with concordant signs. For each structure, left panel: Tdt density versus locomotion; right panel: cFos density versus locomotion. Points are individual animals (Tdt in red; cFos in green). Black line: ordinary-least-squares fit; shaded band: 95% CI (for visualization only). Panel titles report Spearman’s ρ, uncorrected p, and FDR-adjusted q. Densities were computed as counts/area (mm2). Non-selection reflects either lack of FDR significance in one modality and/or opposite correlation directions between Tdt and cFos. Raw data underlying the Figure is shown in S1 and S3 Data. (TIFF) [file pbio.3003622.s003.tiff]

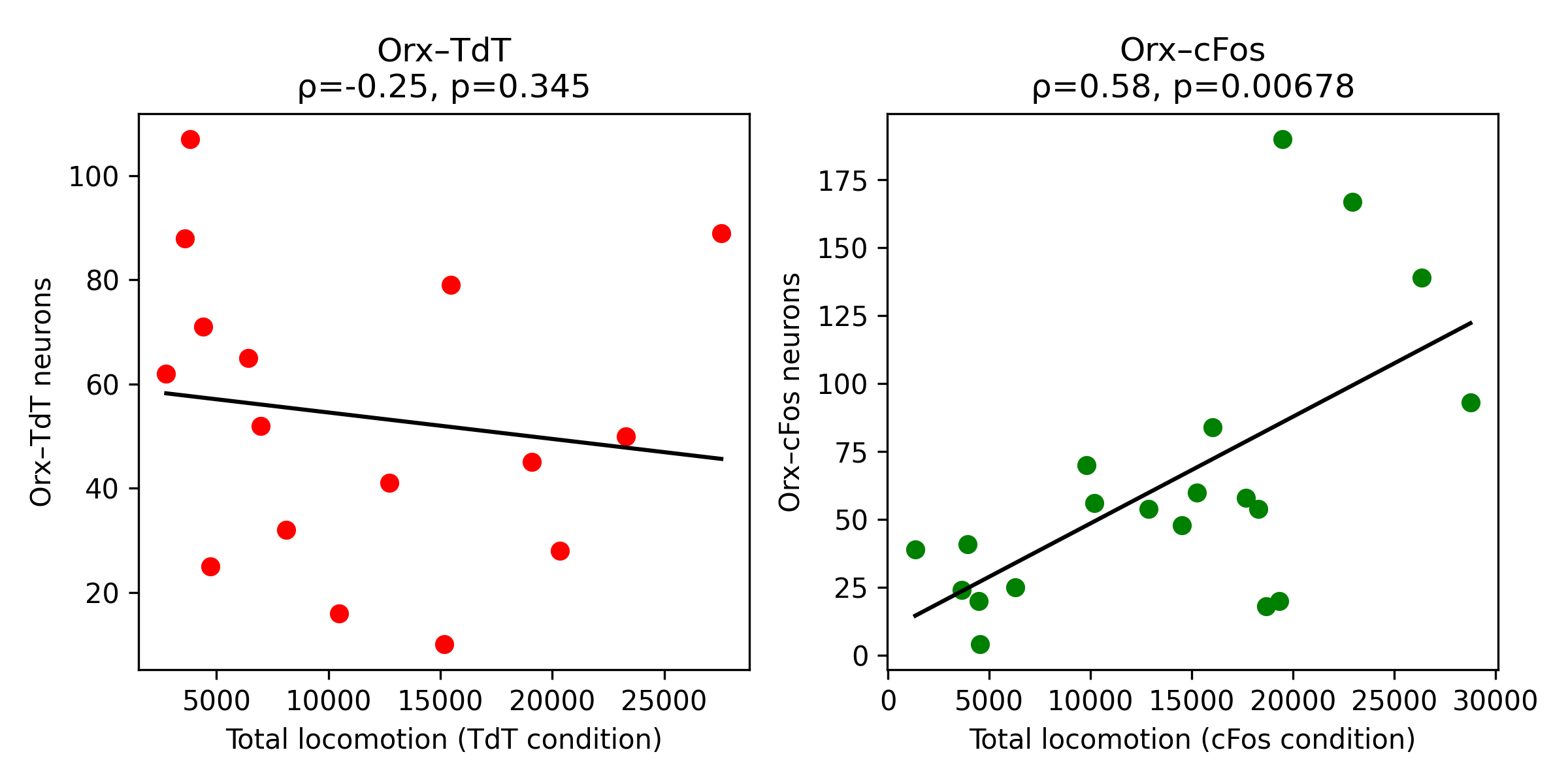

Supplement: S4 Fig — Scatter plots illustrate the relationship between total locomotion and the number of orexin (Orx) neurons labeled with TdTomato (Orx–tdT, left panel, red dots) or expressing cFos (Orx–cFos, right panel, green dots). Each dot represents one animal. Black lines indicate linear regression fits shown for visualization purposes only. Spearman’s rank correlation coefficient (ρ) and associated p-values are indicated in each panel. Locomotor activity was not significantly correlated with Orx–tdT neuron counts, whereas a significant positive correlation was observed between locomotion and Orx–cFos labeling, partly suggesting increased acute recruitment of Orx neurons in animals with higher locomotor activity Raw data underlying the Figure is shown in S1 and S5 Data. (TIFF) [file pbio.3003622.s004.tiff]
