# Supplementary material for: Pharmacological and non-pharmacological methods of inducing wakefulness activate distinct neural populations in the mouse brain
Source: PLoS Biol. 2026 Mar 19;24(3):e3003622. doi: 10.1371/journal.pbio.3003622 (PMC13038112; doi:10.1371/journal.pbio.3003622)
Supplement: S1 Table — For each of the nine structures classified as Sol-specific, the table reports density (cells per unit area; see Methods) of tdt+ and cFos+ neurons in Sol, Mod, NWday, and Nwnight as mean ± SEM, with n (animals) shown beneath each value. (Note: for Nwnight, only cFos is available; tdt is not acquired in that condition. Within each structure and marker (tdt, cFos), densities were modeled with generalized linear models (Gamma family, log link) using the animal as the experimental unit and robust (HC0) standard errors. The table lists planned directional contrasts testing Sol > Mod, Sol > Nwday, and Sol > Nwnight (where applicable), reported as one-sided Wald p-values. P-values are unadjusted and correspond to these targeted, pre-specified comparisons. Raw data underlying the Figure is shown in S3 Data. (DOCX) [file pbio.3003622.s005.docx]

| **Structure** | **Tdt density** | | | **cFos density** | | | | **GLM one-sided p-values** | | | | |
| --- | --- | --- | --- | --- | --- | --- | --- | --- | --- | --- | --- | --- |
|  | **Sol** | **Mod** | **NWday** | **Sol** | **Mod** | **NWday** | **NWnight** | **Tdt S>Mod** | **Tdt S>NWday** | **cFos S>Mod** | **cFos S>NWday** | **cFos S>NWnight** |
| Area postrema | **774.44** ± 173.34  n=8 | **221.93** ± 57.66  n=4 | **415.72** ± 56.25  n=7 | **778.23** ± 185.63  n=7 | **179.07** ± 109.36  n=4 | **143.98** ± 17.76  n=4 | **32.54** ± 15.63  n=4 | <0.001 | 0.005 | 0.005 | <0.001 | <0.001 |
| Bed nuclei of the stria terminalis | **279.34** ± 34.04  n=8 | **146.98** ± 13.49  n=4 | **180.36** ± 10.54  n=9 | **300.34** ± 69.50  n=8 | **60.19** ± 29.79  n=4 | **132.58** ± 24.94  n=4 | **94.80** ± 6.79  n=5 | <0.001 | <0.001 | <0.001 | 0.001 | <0.001 |
| Bed nuclei of the stria terminalis, anterior division, oval nucleus | **784.13** ± 106.67  n=8 | **344.92** ± 99.27  n=4 | **547.93** ± 89.18  n=9 | **1127.83** ± 209.51  n=8 | **197.01** ± 163.45  n=4 | **250.96** ± 82.91  n=4 | **176.75** ± 47.49  n=5 | 0.002 | 0.036 | 0.009 | <0.001 | <0.001 |
| Central amygdalar nucleus, lateral part | **578.86** ± 71.86  n=8 | **200.83** ± 59.78  n=4 | **363.64** ± 37.43  n=9 | **607.48** ± 116.35  n=8 | **140.52** ± 18.83  n=4 | **184.46** ± 11.10  n=4 | **176.31** ± 10.22  n=5 | <0.001 | 0.001 | <0.001 | <0.001 | <0.001 |
| Lateral parabrachial nucleus, external part | **1211.63** ± 85.56  n=8 | **601.76** ± 128.16  n=4 | **565.20** ± 70.37  n=9 | **1464.96** ± 116.40  n=8 | **567.55** ± 250.25  n=4 | **389.29** ± 113.64  n=4 | **262.89** ± 74.48  n=5 | <0.001 | <0.001 | 0.007 | <0.001 | <0.001 |
| Nucleus of the solitary tract, caudal part | **830.34** ± 102.13  n=8 | **325.73** ± 56.18  n=4 | **425.25** ± 32.46  n=8 | **884.36** ± 214.51  n=8 | **281.04** ± 111.97  n=4 | **215.08** ± 33.58  n=4 | **67.27** ± 5.47  n=4 | <0.001 | <0.001 | 0.003 | <0.001 | <0.001 |
| Paraventricular hypothalamic nucleus | **1773.49** ± 185.72  n=8 | **1024.20** ± 68.91  n=4 | **1156.48** ± 173.46  n=9 | **2090.63** ± 281.45  n=8 | **307.50** ± 151.79  n=4 | **373.20** ± 61.43  n=4 | **588.15** ± 228.30  n=5 | <0.001 | 0.006 | <0.001 | <0.001 | <0.001 |
| Paraventricular nucleus of the thalamus | **1002.10** ± 108.65  n=8 | **650.58** ± 63.74  n=4 | **797.18** ± 46.39  n=9 | **838.54** ± 74.39  n=8 | **412.90** ± 96.31  n=4 | **660.36** ± 52.64  n=4 | **338.08** ± 62.72  n=5 | <0.001 | 0.024 | <0.001 | 0.013 | <0.001 |
| Supraoptic nucleus | **1840.03** ± 113.23  n=8 | **973.01** ± 103.77  n=4 | **1098.53** ± 63.72  n=9 | **2032.68** ± 295.62  n=8 | **126.15** ± 56.72  n=4 | **114.81** ± 42.80  n=4 | **105.58** ± 18.93  n=5 | <0.001 | <0.001 | <0.001 | <0.001 | <0.001 |
