# Supplementary material for: Pharmacological and non-pharmacological methods of inducing wakefulness activate distinct neural populations in the mouse brain
Source: PLoS Biol. 2026 Mar 19;24(3):e3003622. doi: 10.1371/journal.pbio.3003622 (PMC13038112; doi:10.1371/journal.pbio.3003622)
Supplement: S2 Table — For each structure, the table reports the RI = (cFostdt neurons)/tdt neurons) as mean ± SEM for the four paired-design groups Mod-Sol, Sol-Mod, NWday-Sol, Sol-Nwday (n is the number of animals contributing to RI for each structure).Planned between-group RI comparisons are shown as two-sided Wald p-values from a binomial GLM (logit link) with robust (HC0) standard errors and frequency weights = tdt counts: Mod-Sol versus Sol-Mod, Nwday-Sol versus Sol-Nwday, Mod-Sol versus Nwday-Sol, and Sol-Mod versus Sol-Nwday. Numeric p-values are reported. Raw data underlying the Figure is shown in S3 Data. (DOCX) [file pbio.3003622.s006.docx]

| **Structure** | **RI (cfos–tdt / tdt)** | | | | **GLM two-sided p-values** | | | |
| --- | --- | --- | --- | --- | --- | --- | --- | --- |
|  | **Mod-Sol** | **Sol-Mod** | **NWday-Sol** | **Sol-NWday** | **Mod-Sol vs Sol-Mod** | **NWday-Sol vs Sol-NWday** | **Mod-Sol vs NWday-Sol** | **Sol-Mod vs Sol-NWday** |
| Area postrema | **0.29** ± 0.09  n=4 | **0.04** ± 0.02  n=4 | **0.21** ± 0.09  n=3 | **0.09** ± 0.05  n=4 | <0.001 | 0.134 | 0.45 | 0.143 |
| Bed nuclei of the stria terminalis | **0.28** ± 0.09  n=4 | **0.08** ± 0.04  n=4 | **0.4** ± 0.04  n=4 | **0.2** ± 0.04  n=4 | 0.006 | <0.001 | 0.175 | 0.025 |
| Bed nuclei of the stria terminalis, anterior division, oval nucleus | **0.49** ± 0.12  n=3 | **0.05** ± 0.05  n=4 | **0.6** ± 0.07  n=4 | **0.14** ± 0.06  n=4 | 0.001 | <0.001 | 0.365 | 0.231 |
| Central amygdalar nucleus, lateral part | **0.47** ± 0.04  n=4 | **0.08** ± 0.02  n=4 | **0.44** ± 0.06  n=4 | **0.1** ± 0.01  n=4 | <0.001 | <0.001 | 0.664 | 0.252 |
| Lateral parabrachial nucleus, external part | **0.59** ± 0.05  n=4 | **0.17** ± 0.06  n=4 | **0.46** ± 0.06  n=4 | **0.13** ± 0.04  n=4 | <0.001 | <0.001 | 0.056 | 0.534 |
| Nucleus of the solitary tract, caudal part | **0.43** ± 0.05  n=4 | **0.13** ± 0.06  n=4 | **0.53** ± 0.06  n=4 | **0.08** ± 0.02  n=4 | 0.001 | <0.001 | 0.124 | 0.373 |
| Paraventricular hypothalamic nucleus | **0.46** ± 0.08  n=4 | **0.09** ± 0.03  n=4 | **0.35** ± 0.13  n=4 | **0.11** ± 0.05  n=4 | <0.001 | 0.024 | 0.408 | 0.725 |
| Paraventricular nucleus of the thalamus | **0.41** ± 0.07  n=4 | **0.18** ± 0.05  n=4 | **0.41** ± 0.03  n=4 | **0.31** ± 0.08  n=4 | 0.003 | 0.216 | 0.995 | 0.088 |
| Supraoptic nucleus | **0.43** ± 0.09  n=4 | **0.03** ± 0.01  n=4 | **0.49** ± 0.06  n=4 | **0.02** ± 0.01  n=4 | <0.001 | <0.001 | 0.588 | 0.682 |
