# Supplementary material for: Pharmacological and non-pharmacological methods of inducing wakefulness activate distinct neural populations in the mouse brain
Source: PLoS Biol. 2026 Mar 19;24(3):e3003622. doi: 10.1371/journal.pbio.3003622 (PMC13038112; doi:10.1371/journal.pbio.3003622)
Supplement: S3 Table — Binomial GLMs were fitted on raw counts. For TdT_TH and cFos_TH contrasts (‘X vs Sol’), OR < 1 indicates a higher proportion in Sol than in X; OR > 1 indicates a lower proportion in Sol. For triple reactivation, the contrast tests Sol_second versus Sol_first within each mirror pair. Q-FDR values are Benjamini–Hochberg corrected within each analysis family. Raw data underlying the Figure is shown in S4 Data. (DOCX) [file pbio.3003622.s007.docx]

# Supplementary Table — TH structures (all results)

Binomial GLMs were fitted on raw counts. For TdT_TH and cFos_TH contrasts ('X vs Sol'), OR < 1 indicates a higher proportion in Sol than in X; OR > 1 indicates a lower proportion in Sol. For triple reactivation, the contrast tests Sol_second vs Sol_first within each mirror pair. q-FDR values are Benjamini–Hochberg corrected within each analysis family.

| Structure | Measure | Contrast | OR | 95% CI | q-FDR | Sig. |
| --- | --- | --- | --- | --- | --- | --- |
| Arcuate hypothalamic nucleus | TdT_TH (tdtth/th) | Mod vs Sol | 0.41 | [0.27, 0.64] | 0.000157 | Yes |
| Arcuate hypothalamic nucleus | TdT_TH (tdtth/th) | NWday vs Sol | 0.39 | [0.29, 0.51] | 1.11e-10 | Yes |
| Arcuate hypothalamic nucleus | Triple reactivation (fostdtth/tdtth) | Mod: Sol_second vs Sol_first | 6.53 | [2.24, 19.08] | 0.0018 | Yes |
| Arcuate hypothalamic nucleus | Triple reactivation (fostdtth/tdtth) | NWday: Sol_second vs Sol_first | 3.48 | [1.68, 7.22] | 0.00238 | Yes |
| Arcuate hypothalamic nucleus | cFos_TH (fosth/th) | Mod vs Sol | 0.20 | [0.11, 0.34] | 6.06e-08 | Yes |
| Arcuate hypothalamic nucleus | cFos_TH (fosth/th) | NWday vs Sol | 0.52 | [0.33, 0.82] | 0.00904 | Yes |
| Arcuate hypothalamic nucleus | cFos_TH (fosth/th) | NWnight vs Sol | 0.03 | [0.01, 0.10] | 1.64e-08 | Yes |
| Area postrema | TdT_TH (tdtth/th) | Mod vs Sol | 0.55 | [0.25, 1.24] | 0.179 | No |
| Area postrema | TdT_TH (tdtth/th) | NWday vs Sol | 0.25 | [0.11, 0.57] | 0.002 | Yes |
| Area postrema | Triple reactivation (fostdtth/tdtth) | Mod: Sol_second vs Sol_first | 2451971849.08 | [0.00, inf] | 0.999 | No |
| Area postrema | Triple reactivation (fostdtth/tdtth) | NWday: Sol_second vs Sol_first | 6314210235.77 | [0.00, inf] | 0.999 | No |
| Area postrema | cFos_TH (fosth/th) | Mod vs Sol | 0.16 | [0.05, 0.56] | 0.00723 | Yes |
| Area postrema | cFos_TH (fosth/th) | NWday vs Sol | 0.00 | [0.00, inf] | 0.999 | No |
| Area postrema | cFos_TH (fosth/th) | NWnight vs Sol | 0.00 | [0.00, inf] | 0.999 | No |
| Dorsal nucleus raphe | TdT_TH (tdtth/th) | Mod vs Sol | 0.68 | [0.49, 0.94] | 0.0286 | Yes |
| Dorsal nucleus raphe | TdT_TH (tdtth/th) | NWday vs Sol | 0.47 | [0.35, 0.63] | 1.47e-06 | Yes |
| Dorsal nucleus raphe | Triple reactivation (fostdtth/tdtth) | Mod: Sol_second vs Sol_first | 1.70 | [0.60, 4.82] | 0.475 | No |
| Dorsal nucleus raphe | Triple reactivation (fostdtth/tdtth) | NWday: Sol_second vs Sol_first | 2.03 | [0.89, 4.65] | 0.141 | No |
| Dorsal nucleus raphe | cFos_TH (fosth/th) | Mod vs Sol | 0.32 | [0.17, 0.58] | 0.000368 | Yes |
| Dorsal nucleus raphe | cFos_TH (fosth/th) | NWday vs Sol | 1.43 | [0.97, 2.11] | 0.0966 | No |
| Dorsal nucleus raphe | cFos_TH (fosth/th) | NWnight vs Sol | 0.38 | [0.22, 0.67] | 0.00184 | Yes |
| Lateral reticular nucleus | TdT_TH (tdtth/th) | Mod vs Sol | 0.34 | [0.24, 0.49] | 2.28e-08 | Yes |
| Lateral reticular nucleus | TdT_TH (tdtth/th) | NWday vs Sol | 0.26 | [0.19, 0.36] | 4.3e-16 | Yes |
| Lateral reticular nucleus | Triple reactivation (fostdtth/tdtth) | Mod: Sol_second vs Sol_first | 12.31 | [5.60, 27.05] | 3.67e-09 | Yes |
| Lateral reticular nucleus | Triple reactivation (fostdtth/tdtth) | NWday: Sol_second vs Sol_first | 11.11 | [2.35, 52.53] | 0.00535 | Yes |
| Lateral reticular nucleus | cFos_TH (fosth/th) | Mod vs Sol | 0.25 | [0.17, 0.37] | 7.91e-11 | Yes |
| Lateral reticular nucleus | cFos_TH (fosth/th) | NWday vs Sol | 0.38 | [0.25, 0.57] | 9.88e-06 | Yes |
| Lateral reticular nucleus | cFos_TH (fosth/th) | NWnight vs Sol | 0.01 | [0.00, 0.02] | 4.76e-12 | Yes |
| Locus ceruleus | TdT_TH (tdtth/th) | Mod vs Sol | 0.28 | [0.20, 0.39] | 7.61e-13 | Yes |
| Locus ceruleus | TdT_TH (tdtth/th) | NWday vs Sol | 0.33 | [0.26, 0.41] | 1.68e-22 | Yes |
| Locus ceruleus | Triple reactivation (fostdtth/tdtth) | Mod: Sol_second vs Sol_first | 4.14 | [1.45, 11.81] | 0.0141 | Yes |
| Locus ceruleus | Triple reactivation (fostdtth/tdtth) | NWday: Sol_second vs Sol_first | 14.18 | [5.05, 39.81] | 4.27e-06 | Yes |
| Locus ceruleus | cFos_TH (fosth/th) | Mod vs Sol | 0.21 | [0.12, 0.38] | 8.17e-07 | Yes |
| Locus ceruleus | cFos_TH (fosth/th) | NWday vs Sol | 0.64 | [0.42, 0.97] | 0.0528 | No |
| Locus ceruleus | cFos_TH (fosth/th) | NWnight vs Sol | 0.01 | [0.00, 0.08] | 1.76e-05 | Yes |
| Nucleus of the solitary tract | TdT_TH (tdtth/th) | Mod vs Sol | 0.62 | [0.43, 0.90] | 0.0209 | Yes |
| Nucleus of the solitary tract | TdT_TH (tdtth/th) | NWday vs Sol | 0.59 | [0.44, 0.79] | 0.000988 | Yes |
| Nucleus of the solitary tract | Triple reactivation (fostdtth/tdtth) | Mod: Sol_second vs Sol_first | 8.73 | [3.88, 19.60] | 6.99e-07 | Yes |
| Nucleus of the solitary tract | Triple reactivation (fostdtth/tdtth) | NWday: Sol_second vs Sol_first | 9.09 | [3.51, 23.51] | 2.39e-05 | Yes |
| Nucleus of the solitary tract | cFos_TH (fosth/th) | Mod vs Sol | 0.14 | [0.09, 0.21] | 2.13e-18 | Yes |
| Nucleus of the solitary tract | cFos_TH (fosth/th) | NWday vs Sol | 0.22 | [0.14, 0.34] | 3.11e-10 | Yes |
| Nucleus of the solitary tract | cFos_TH (fosth/th) | NWnight vs Sol | 0.02 | [0.00, 0.08] | 1.99e-07 | Yes |
| Periventricular zone | TdT_TH (tdtth/th) | Mod vs Sol | 0.45 | [0.32, 0.65] | 3.28e-05 | Yes |
| Periventricular zone | TdT_TH (tdtth/th) | NWday vs Sol | 0.73 | [0.56, 0.95] | 0.0286 | Yes |
| Periventricular zone | Triple reactivation (fostdtth/tdtth) | Mod: Sol_second vs Sol_first | 1.11 | [0.47, 2.65] | 0.999 | No |
| Periventricular zone | Triple reactivation (fostdtth/tdtth) | NWday: Sol_second vs Sol_first | 1.80 | [0.92, 3.53] | 0.141 | No |
| Periventricular zone | cFos_TH (fosth/th) | Mod vs Sol | 0.49 | [0.35, 0.71] | 0.000259 | Yes |
| Periventricular zone | cFos_TH (fosth/th) | NWday vs Sol | 0.88 | [0.64, 1.21] | 0.554 | No |
| Periventricular zone | cFos_TH (fosth/th) | NWnight vs Sol | 0.94 | [0.66, 1.35] | 0.847 | No |
| Substantia nigra, compact part | TdT_TH (tdtth/th) | Mod vs Sol | 0.59 | [0.06, 5.71] | 0.686 | No |
| Substantia nigra, compact part | TdT_TH (tdtth/th) | NWday vs Sol | 0.69 | [0.14, 3.41] | 0.686 | No |
| Substantia nigra, compact part | cFos_TH (fosth/th) | Mod vs Sol | 0.00 | [0.00, inf] | 0.999 | No |
| Substantia nigra, compact part | cFos_TH (fosth/th) | NWday vs Sol | 0.00 | [0.00, inf] | 0.999 | No |
| Substantia nigra, compact part | cFos_TH (fosth/th) | NWnight vs Sol | 1.93 | [0.17, 21.28] | 0.711 | No |
| Ventral tegmental area | TdT_TH (tdtth/th) | Mod vs Sol | 0.08 | [0.01, 0.62] | 0.0252 | Yes |
| Ventral tegmental area | TdT_TH (tdtth/th) | NWday vs Sol | 0.58 | [0.34, 1.00] | 0.0602 | No |
| Ventral tegmental area | Triple reactivation (fostdtth/tdtth) | Mod: Sol_second vs Sol_first | 2563606287.88 | [0.00, inf] | 0.999 | No |
| Ventral tegmental area | Triple reactivation (fostdtth/tdtth) | NWday: Sol_second vs Sol_first | 1.73 | [0.34, 8.87] | 0.654 | No |
| Ventral tegmental area | cFos_TH (fosth/th) | Mod vs Sol | 0.07 | [0.01, 0.55] | 0.0177 | Yes |
| Ventral tegmental area | cFos_TH (fosth/th) | NWday vs Sol | 0.59 | [0.29, 1.19] | 0.182 | No |
| Ventral tegmental area | cFos_TH (fosth/th) | NWnight vs Sol | 0.36 | [0.21, 0.61] | 0.000296 | Yes |
| Zona incerta | TdT_TH (tdtth/th) | Mod vs Sol | 1.05 | [0.55, 2.00] | 0.887 | No |
| Zona incerta | TdT_TH (tdtth/th) | NWday vs Sol | 0.55 | [0.32, 0.96] | 0.0453 | Yes |
| Zona incerta | Triple reactivation (fostdtth/tdtth) | Mod: Sol_second vs Sol_first | 7.50 | [1.78, 31.68] | 0.0138 | Yes |
| Zona incerta | Triple reactivation (fostdtth/tdtth) | NWday: Sol_second vs Sol_first | 0.00 | [0.00, inf] | 0.999 | No |
| Zona incerta | cFos_TH (fosth/th) | Mod vs Sol | 0.51 | [0.28, 0.94] | 0.0456 | Yes |
| Zona incerta | cFos_TH (fosth/th) | NWday vs Sol | 0.37 | [0.16, 0.87] | 0.035 | Yes |
| Zona incerta | cFos_TH (fosth/th) | NWnight vs Sol | 0.22 | [0.11, 0.48] | 0.000259 | Yes |
