# Supplementary material for: Pharmacological and non-pharmacological methods of inducing wakefulness activate distinct neural populations in the mouse brain
Source: PLoS Biol. 2026 Mar 19;24(3):e3003622. doi: 10.1371/journal.pbio.3003622 (PMC13038112; doi:10.1371/journal.pbio.3003622)
Supplement: S4 Table — Odds ratios (OR) with 95% confidence intervals are reported from binomial GLMs. For contrasts of the form ‘X vs Sol’, OR < 1 indicates higher labeling in Sol. For triple reactivation tests, OR > 1 indicates higher reactivation when Sol is the second episode. P-values are FDR-corrected across all planned tests. Raw data underlying the Figure is shown in S5 Data. (DOCX) [file pbio.3003622.s008.docx]

# Orexin neurons (LHA): Summary of planned statistical tests

Odds ratios (OR) with 95% confidence intervals are reported from binomial GLMs. For contrasts of the form 'X vs Sol', OR < 1 indicates higher labeling in Sol. For triple reactivation tests, OR > 1 indicates higher reactivation when Sol is the second episode. P-values are FDR-corrected across all planned tests.

| Measure | Contrast | OR | 95% CI | p | q-FDR | Interpretation |
| --- | --- | --- | --- | --- | --- | --- |
| TdT/Orx (double; Condition1) | Mod vs Sol | 1.18 | [0.94, 1.48] | 0.153 | 0.178 | Sol lower |
| TdT/Orx (double; Condition1) | NWday vs Sol | 1.67 | [1.42, 1.95] | 2.29e-10 | 8.01e-10 | Sol lower |
| cFos/Orx (double; Condition2) | Mod vs Sol | 1.46 | [1.18, 1.82] | 0.000632 | 0.00148 | Sol lower |
| cFos/Orx (double; Condition2) | NWday vs Sol | 1.14 | [0.88, 1.46] | 0.32 | 0.32 | Sol lower |
| cFos/Orx (double; Condition2) | NWnight vs Sol | 2.56 | [2.13, 3.06] | 2.6e-24 | 1.82e-23 | Sol lower |
| Triple reactivation (Fos+TdT+Orx among TdT+Orx) | Mod: Sol_second vs Sol_first | 0.63 | [0.44, 0.89] | 0.00897 | 0.0126 | Sol higher |
| Triple reactivation (Fos+TdT+Orx among TdT+Orx) | NWday: Sol_second vs Sol_first | 0.48 | [0.31, 0.74] | 0.000848 | 0.00148 | Sol higher |
