# Supplementary material for: Pharmacological and non-pharmacological methods of inducing wakefulness activate distinct neural populations in the mouse brain
Source: PLoS Biol. 2026 Mar 19;24(3):e3003622. doi: 10.1371/journal.pbio.3003622 (PMC13038112; doi:10.1371/journal.pbio.3003622)
Supplement: S5 Table — Structures with averaged reactivation index above 0.3 in all groups and with a p > 0.05 in one-way ANOVA (d.f. = 3,12), excepting the Lateral Reticular Nucleus showing just a value just above (0.0051). The numbers in each group show the mean ± SEM. Raw data underlying the Figure is shown in S3 Data. (DOCX) [file pbio.3003622.s009.docx]

| **Structure** | **Sol-Mod** | **Sol-NWday** | **Mod-Sol** | **NWday-Sol** | **NWday-NWnight** |
| --- | --- | --- | --- | --- | --- |
| Geniculate group, ventral thalamus | 0.50 ± 0.08 | 0.61 ± 0.08 | 0.51 ± 0.12 | 0.58 ± 0.08 | 0.45 ± 0.01 |
| Parasubthalamic nucleus | 0.43 ± 0.08 | 0.58 ± 0.04 | 0.57 ± 0.05 | 0.48 ± 0.07 | 0.36 ± 0.03 |
| Tegmental reticular nucleus | 0.44 ± 0.09 | 0.54 ± 0.09 | 0.49 ± 0.13 | 0.29 ± 0.06 | 0.33 ± 0.03 |
| Inferior colliculus | 0.34 ± 0.04 | 0.49 ± 0.05 | 0.43 ± 0.05 | 0.46 ± 0.07 | 0.37 ± 0.03 |
| Laterodorsal tegmental nucleus | 0.38 ± 0.03 | 0.44 ± 0.05 | 0.43 ± 0.04 | 0.53 ± 0.05 | 0.23 ± 0.03 |
| Superior colliculus, sensory related | 0.37 ± 0.07 | 0.44 ± 0.00 | 0.34 ± 0.09 | 0.39 ± 0.04 | 0.43 ± 0.02 |
| Posterior hypothalamic nucleus | 0.31 ± 0.06 | 0.52 ± 0.06 | 0.45 ± 0.05 | 0.40 ± 0.10 | 0.27 ± 0.03 |
| Supramammillary nucleus | 0.34 ± 0.12 | 0.37 ± 0.11 | 0.48 ± 0.11 | 0.48 ± 0.05 | 0.24 ± 0.01 |
| Superior colliculus, motor related | 0.26 ± 0.06 | 0.50 ± 0.10 | 0.28 ± 0.07 | 0.43 ± 0.03 | 0.33 ± 0.02 |
| Lateral reticular nucleus | 0.37 ± 0.04 | 0.30 ± 0.05 | 0.52 ± 0.02 | 0.37 ± 0.03 | 0.21 ± 0.02 |
| Lateral hypothalamic area | 0.27 ± 0.04 | 0.47 ± 0.08 | 0.40 ± 0.06 | 0.38 ± 0.05 | 0.25 ± 0.02 |
| Nucleus of the solitary tract, rostral part | 0.29 ± 0.05 | 0.45 ± 0.10 | 0.33 ± 0.10 | 0.41 ± 0.11 | 0.25 ± 0.04 |
| Pretectal region | 0.27 ± 0.06 | 0.42 ± 0.03 | 0.34 ± 0.04 | 0.40 ± 0.04 | 0.26 ± 0.01 |
| Periaqueductal gray | 0.29 ± 0.02 | 0.39 ± 0.04 | 0.29 ± 0.07 | 0.46 ± 0.04 | 0.23 ± 0.02 |
| Dorsal premammillary nucleus | 0.34 ± 0.03 | 0.33 ± 0.00 | 0.38 ± 0.14 | 0.23 ± 0.00 | 0.27 ± 0.03 |
| Ventral premammillary nucleus | 0.24 ± 0.02 | 0.26 ± 0.07 | 0.37 ± 0.02 | 0.35 ± 0.12 | 0.21 ± 0.06 |
| Basomedial amygdalar nucleus | 0.21 ± 0.04 | 0.32 ± 0.02 | 0.25 ± 0.02 | 0.24 ± 0.03 | 0.22 ± 0.02 |
