# Supplementary material for: Pharmacological and non-pharmacological methods of inducing wakefulness activate distinct neural populations in the mouse brain
Source: PLoS Biol. 2026 Mar 19;24(3):e3003622. doi: 10.1371/journal.pbio.3003622 (PMC13038112; doi:10.1371/journal.pbio.3003622)
Supplement: Attachment — Submitted filename: ResponsetoReviewerPLoSBiol.docx [file pbio.3003622.s015.docx]

Dear Editor,

Please find enclosed our revised manuscript named: Drug-induced versus non-pharmacological wakefulness: similar or different states? A whole brain analysis in TRAP2 transgenic mice.

We addressed your request as well as those of the reviewers in the new version. Below I detail all the modifications made to the manuscript:

*Reviewer remarks:*

*1) there is no comparison of cFos immunostaining vs. TdTom TRAP labeling for the same condition (e.g., modafinil vs modafinil). The authors seem to be interpreting all of their findings as indicating different treatments activate different neurons, but actually the two different techniques NEVER consistently label the same neurons (even for identical conditions). Therefore, a critical positive control is missing here. It is hard to make any real conclusions based on the groups presented - where two variables (condition and method) have been mixed together.*

*2) the N for these studies (3-4 measures per condition) is far too small to have confidence in the results. The statistical tests are almost certainly underpowered. Also, the use of quartile box plots is a little misleading when there are only 3-4 measures.*

The reviewer found that the N is not large enough for having confidence in the results. However, he did not take into account that we have four groups of mice with cFos and Tdtomato labeling meaning that we have the values from 16 mice in the Sol condition (8 cFos and 8 tdT) and eight mice in the Mod or NWday conditions (4 cFos and 4 tdT). In addition, we have the double staining of tdT and cFos in the two mirror groups (Sol-Mod, Mod-Sol and Sol-NWday, NWday-Sol) which is key for reaching a solid conclusion. Further, in order to make a more solid selection in the new version of the manuscript, we designed a unique new statistical method crossing all results obtained. We considered that a structure was modified only if the differences in the density of tdT neurons, the difference in the density of cFos neurons and in the number of double-labeled neurons were all significant. We did not anymore consider a structure as modified if only one or two of the above comparisons were not matching. Further, we added subdivisions to several structures showing a larger activation in the Sol condition. Indeed, when reexamining our results we realized that for several structures, the labeled neurons were more numerous in only a specific part of the nuclei as identified by Brain atlases. These sub-structures included the oval part of the bed nucleus of the stria terminalis, the lateral part of the central amygdalar nucleus, the external part of the lateral parabrachial nucleus and the caudal part of the nucleus of the solitary tract. By this means, we were able to reach an unprecedented level of anatomical description and an extremely high level of significance. This was also made possible by using new up to date statistical generalized linear models (GLMs).

In summary, all the modifications made to our analysis allowed us to reach a new standard for analysing complex data obtained by the TRAP2 mice. We believe that such a method of analysis will be largely used in the future to solidly demonstrate modifications of neuronal activation such as those reported here.

*3) the quantification of behavior during wake is minimal to nonexistent. Measures of locomotor activity etc., need to be compared between groups. Also there is no quantification or detailed description of interventions for sensory stimulation-driven NW*

We fully agree on that point and we did extensive analysis of the video recordings and of course EEG and EMG recordings. We now report in Fig. 2 the spontaneous locomotor activity of all groups of mice. We also report in Fig. 3 the spectral characteristics of wakefulness induced by solriamfetol and modafinil compared to non-pharmacological wake. Our new analysis shows that solriamfetol treatment induced a decrease in activity in mice compared to modafinil and NWday and night. Further, we show that the density of cFos and tdT labeled neurons is inversely correlated with locomotor activity for most of the structures showing a higher activation in the Sol condition. We finally show in Figure 3a that solriamfetol treatment decreased theta peak frequency.

*4) the use of just one dose of drug is not justified and scientifically suboptimal. This makes it even more difficult to make any conclusions from the data.*

For our study, we needed to be able to compare the level of activation induced by two different drugs with non-pharmacological wake. To this aim, it was needed to obtain a similar duration of wakefulness. We decided that 2h of wake was necessary to obtain a maximal staining of tdT and cFos. We therefore look at previous studies to choose doses of Mod and Sol inducing two hours of continuous wakefulness. We then tested whether the doses selected were effectively inducing two hours of wakefulness. Since it was the case we did stick with these doses for the entire study since higher or lower doses would not be adapted to a functional neuroanatomical study.

*Reviewer #2, Shuancheng Ren (note: reviewer 2 has signed this review): In the present study, the authors systemically evaluated the whole brain neuronal activation during drug-induced wakefulness and non-pharmacological wakefulness using TRAP2 mice. By exploring a variety of rigorous and impressive analysis methods, they found that substantial structures were activated in solriamfetol-, modafinil-, or non-pharmacological wakefulness, with more structures were activated by solriamfetol than modafinil or non-pharmacological wakefulness. Importantly, they also revealed that many structures not previously involved in wakefulness might play a key role in regulating the wakefulness state. These results not only provide mechanisms underlying drug-induced wakefulness, but also shed light on discovering potential novel wakefulness-promoting neurons using present methodological strategies. The results were clearly presented and the manuscript was well-written.*

We thank the reviewer for these very positive comments

*However, there are some concerns need to be further addressed, which may improve the impact of present study.*

*1. Solriamfetol, modafinil, or non-pharmacological wakefulness induced different pattern of neuronal activation. Does the author investigate why these three conditions have different effects? Considering that these three methods differentially affect wakefulness as shown in figure 1b and figure 1c, a correlative analysis between time in wakefulness and pattern of neuronal activation may give some clues. In addition, as arousal level is dynamically changed during wakefulness state, which can be measured by locomotor activities, EEG theta activity, or pupil size. Though these three methods induced similar wakefulness time in certain duration, they may have different effects on arousal level. This may also contribute to the different pattern of neuronal activation. A further analysis the relationship between arousal level and neuronal activation may helpful.*

We fully agree on that point and we did extensive analysis of the video recordings and of course EEG and EMG recordings. We now report in Fig. 2 the spontaneous locomotor activity of all groups of mice. We also report in Fig. 3 the spectral characteristics of wakefulness induced by solriamfetol and modafinil compared to non-pharmacological wake. Our new analysis shows that solriamfetol treatment induced a decrease in activity in mice compared to modafinil and NWday and night. Further, we show that the density of cFos and tdT labeled neurons is inversely correlated with locomotor activity for most of the structures showing a higher activation in the Sol condition. We finally show in Figure 3a that solriamfetol treatment decreased theta peak frequency.

*2. The authors compared tdT and cFos in drug and sensory stimulation non-pharmacological wakefulness. Did the author observe tdT and cFos expression in normal wakefulness?*

The control we are using is wake promoted by intermittent sensory stimulations for two (cFos) or four hours (tdtomato) of the mice to keep them continuously awake. The advantage of such control is that it is done at the same circadian time as the Sol and Mod injections. The possible drawback is that part of the labeling might be induced by the sensory stimulation although we stimulate the mice only when they are starting to sleep. When the reviewer is asking for a natural wake, our control is in fact indeed corresponding to a natural wake. However, it is not a wake period without any external stimulation. Indeed, it is impossible to obtain a long wake period during the day since mice are mostly sleeping at that time. The only way to get a longer period of wake was to perfuse mice during the night during which they are more awake although we needed to be sure that they stay awake for two hours and it did imply some sensory stimulation. Such control was also very important for two aspects the reviewer did not mention. It allowed us to eliminate from our listing circadian structures activated during the day by the light or structures activated by the sleep deprivation induced. Therefore, we added a group of four Wday-Wnight mice to the study. We now provide analysis of these mice and compare the results obtained between the two conditions as well as with the previous groups of mice. We show that the results obtained with the Wnight condition are for nearly all structures not significantly different that those obtained in the Wday condition indicating that the two conditions are similar and represent a state of natural wake.

*3. I am very interested in the structures identified in the present study, such as medial and median preoptic nuclei, periventricular region, supraoptic nucleus (SON), tuberal nucleus, nucleus of the solitary tract (NTS), area postrema and lateral reticular nucleus, which have not been implicated in wakefulness control. Did the authors investigate one or more structures in wakefulness regulation? There results will increase the power of present strategies for mapping novel wakefulness-promoting neurons.*

We agree with the reviewer that chemo and optogenetic experiments would be a nice addition to the paper. However, it would be very time consuming to add them to this paper. It would delay for several years its publication. We therefore choose to make a very complete and serious neuroanatomical analysis to provide a solid ground for future research on the Wake-inducing systems. Our results are also supported by recent new data showing that several new structures identified play a key role in induction of wakefulness.

*Reviewer #3: In this manuscript, Maciel et al. use TRAP2 mouse model to examine whole-brain activation patterns induced by the wake-promoting drugs modafinial and solriamfetol compared to non-pharmacological wakefulness. Solriamfetol strongly activates specific subcortical structures, such as the bed nucleus of the stria terminalis, central amygdala, and paraventricular hypothalamic and thalamic nuclei, more than modafinil or natural wakefulness. However, certain regions, including orexin neurons and the laterodorsal tegmental nucleus, are activated across all wake-induction methods. These findings highlight previously unrecognized brain regions involved in wakefulness and suggest distinct neural mechanisms underlying different wake-promoting strategies, with potential implications for sleep disorders like hypersomnia.*

We thank the reviewer for these very positive comments

*It will be important to characterize wakefulness after drug/natural wake (differences in EMG, EEG changes in different freq such as delta, theta etc) to determine what kind of wakefulness each condition is inducing. Authors should determine whether any of these parameters are correlated with cFos/tdTom activation patterns.*

We fully agree on that point and we did extensive analysis of the video recordings and of course EEG and EMG recordings. We now report in Fig. 2 the spontaneous locomotor activity of all groups of mice. We also report in Fig. 3 the spectral characteristics of wakefulness induced by solriamfetol and modafinil compared to non-pharmacological wake. Our new analysis shows that solriamfetol treatment induced a decrease in activity in mice compared to modafinil and NWday and night. Further, we show that the density of cFos and tdT labeled neurons is inversely correlated with locomotor activity for most of the structures showing a higher activation in the Sol condition. We finally show in Figure 3a that solriamfetol treatment decreased theta peak frequency.

*In addition, it is not clear why the authors chose the specific drug dose as cfos/tdTom activation pattern could depend on the dose (how much/how long wake is induced, or whether EMG activation is larger by higher dose etc).*

For our study, we needed to be able to compare the level of activation induced by two different drugs with non-pharmacological wake. To this aim, it was needed to obtain a similar duration of wakefulness. We decided that 2h of wake was necessary to obtain a maximal staining of tdT and cFos. We therefore look at previous studies to choose doses of Mod and Sol inducing two hours of continuous wakefulness. We then tested whether the doses selected were effectively inducing two hours of wakefulness. Since it was the case we did stick with these doses for the entire study since higher or lower doses would not be adapted to a functional neuroanatomical study.

*Line 787: these drugs and natural wakefulness activate/inhibit neurons differently. Perhaps this is not surprising given that they promote different kinds of wakefulness (EEG frequencies, EMG activation etc). It will be crucial to characterize the wake pattern after drug injections and spontaneous wakefulness and compare/correlate these data sets with cFos/tdTom expression*

We fully agree on that point and we did extensive analysis of the video recordings and of course EEG and EMG recordings. We now report in Fig. 2 the spontaneous locomotor activity of all groups of mice. We also report in Fig. 3 the spectral characteristics of wakefulness induced by solriamfetol and modafinil compared to non-pharmacological wake. Our new analysis shows that solriamfetol treatment induced a decrease in activity in mice compared to modafinil and NWday and night. Further, we show that the density of cFos and tdT labeled neurons is inversely correlated with locomotor activity for most of the structures showing a higher activation in the Sol condition. We finally show in Figure 3a that solriamfetol treatment decreased theta peak frequency.

*Previous study, Hasan 2009 did the same investigation as this manuscript showing cFos activation after drug treatment/natural wakefulness, which reduces the novelty of this study. However Hasan 2009 did not show quantification/sophisticated characterization of all brain regions as shown in this manuscript.*

As stated by the reviewer Hasan work was only preliminary and they did not report the new results provided by our study

*Line30-32: authors should not assume that all the activated neurons (cFos, tdTom) are involved in wakefulness. These drugs affect various neuromodulators/different types of behaviors, and therefore activate a variety of neurons. Therefore, the results from this study does not necessarily point to wakefulness-related neurons, rather neural activation related to specific drugs (these activated neurons may be affected by the pharmacology of specific drugs/receptors/associated behaviors). Therefore, this study will not determine which populations of neurons are involved in the induction of waking by specific drug as authors proposed (line 137-138, 158-159). Authors need to be careful with this interpretation. Without functional studies (activation, inhibition), authors cannot conclude that these cFos/tdTom neurons are crucial for regulating wakefulness.*

We agree with the reviewers as stated above also. Indeed, the main effect of solriamfetol and modafinil is to induce wakefulness but they could have more specific effects and it is not possible to disentangle whether this is the case. Our results showing that the two drugs recruit the same but also different networks is nevertheless crucial since they are both used interchangeably to cure hypersomnia in patients.

*Sulaman 2023 review paper is highly cited throughout the text. While that is a comprehensive review, it would be better to be more specific by citing the most relevant papers as appropriate (not one to fit all).*

We agree with the reviewer and now cite relevant papers as appropriate.

*Fig 2. Authors need to perform separate correlation analysis in different structures (for example, using a different analysis such as linear mixed effects model)*

In the new version of the manuscript, we decided to use generalized linear models (GLMs) because they better fit our data. Our measurements are indeed densities (not well described by a bell curve), and the reactivation index (RI) is a fraction bounded between 0 and 1. GLMs let us model these outcomes with appropriate assumptions, compare conditions fairly even when group sizes differ, and incorporate the RI’s denominator (tdT) directly rather than treating all measurements as equal. In summary, GLMs let us avoid unrealistic normality assumptions and gave clearer, more reliable estimates of how Sol, Mod, NWday, and NWnight differed.

*While authors thoroughly analyze activated neurons, the story is very descriptive, and we cannot understand the function of these activated neurons without functional studies (activation or inactivation studies).*

We agree with the reviewers that chemo and optogenetic experiments would be a nice addition to the paper. However, it would be very time consuming to add them to this paper. It would delay for several years its publication. We prefer to make a very complete and serious neuroanatomical analysis to provide a solid ground for future research on the Wake-inducing systems. Our results are also supported by recent new data showing that several new structures identified play a key role in induction of wakefulness.

*Reviewer #4: This study provides valuable insights into the neural mechanisms underlying wakefulness, utilizing TRAP mice to compare neuronal activation during wakefulness induced by two different drugs—modafinil and solriamfetol—versus sensory stimulation. The results show a strong correlation between tdTomato-labeled neurons and endogenous c-Fos expression, suggesting that these two methods are broadly comparable. The study offers a comprehensive overview of the distinct neural populations activated by pharmacological versus non-pharmacological methods of inducing wakefulness.*

We thank the reviewer for these very positive comments

*1. Categorization of Structures Involved in Wakefulness: The term "More activated" in the abstract lacks the clarity of the other two expressions, "strongly activated" and "very poorly or not strongly activated." The classification of activated structures involved in wakefulness into three distinct groups needs further clarification. The rationale behind this classification should be more thoroughly explained to help readers understand the criteria used. A concise summary of this section should also be provided to improve overall clarity and comprehension.*

In the new version, we designed a more robust method to select the structures modified by the conditions. We have the values from 16 mice in the Sol condition (8 cFos and 8 tdT) and eight mice in the Mod or NWday conditions (4 cFos and 4 tdt). In addition, we have the double staining of tdT and cFos in the two mirror groups (Sol-Mod, Mod-Sol and Sol-NWday, NWday-Sol) which is key for reaching a solid conclusion. Further, in order to make a more solid selection in the new version of the manuscript, we designed a unique new statistical method crossing all results obtained. We considered that a structure was modified only if the differences in the density of tdT neurons, the difference in the density of cfos neurons and in the number of double-labeled neurons were all significant. We did not anymore consider a structure as modified if only one or two of the above comparisons were not matching. Further, we added subdivisions to several structures showing a larger activation in the Sol condition. Indeed, when reexamining our results we realized that for several structures, the labeled neurons were more numerous in only a specific part of the nuclei as identified by Brain atlases. These sub-structures included the oval part of the bed nucleus of the stria terminalis, the lateral part of the central amygdalar nucleus, the external part of the lateral parabrachial nucleus and the caudal part of the nucleus of the solitary tract. By this means, we were able to reach an unprecedented level of anatomical description and an extremely high level of significance. This was also made possible by using new up to date statistical generalized linear models (GLMs).

In summary, all the modifications made to our analysis allowed us to reach a new standard for analysing complex data obtained by the TRAP2 mice. We believe that such a method of analysis will be largely used in the future to solidly demonstrate modifications of neuronal activation such as those reported here.

*2. TRAP Technology vs. Conventional c-Fos Measurements: The comparison between TRAP technology and conventional c-Fos expression measurements should be explicitly detailed in the Methods section. The authors need to clarify the specific methods used to compare these two techniques and validate their reliability and complementarity. Additionally, the significance of TRAP technology in identifying novel regions involved in arousal regulation should be highlighted, as this is a key aspect of the study.*

We now detail the difference in methodology between the TRAP technology and conventional cFos expression in the Methods section. We also clarified the method used to compare the two results obtained by the two methods (see above). A crucial advantage of our approach is that we can determine whether the same neurons are activated in the two conditions which is impossible with classical cFos staining. This constitutes one of the great advances provided by the TRAP method.

*3. Control Group Under Natural Physiological Conditions: The inclusion of a control group representing natural physiological conditions is necessary for direct comparisons between the pharmacologically-induced wakefulness group and the non-pharmacologically-induced wakefulness group. This would strengthen the study's findings and help clarify any differences between these conditions.*

The control we are using is wake promoted by intermittent sensory stimulations for two (cFos) or four hours (tdtomato) of the mice to keep them continuously awake. The advantage of such control is that it is done at the same circadian time as the Sol and Mod injections. The possible drawback is that part of the labeling might be induced by the sensory stimulation although we stimulate the mice only when they are starting to sleep. When the reviewer is asking for a natural wake, our control is in fact indeed corresponding to a natural wake. However, it is not a wake period without any external stimulation. Indeed, it is impossible to obtain a long wake period during the day since mice are mostly sleeping at that time. The only way to get a longer period of wake was to perfuse mice during the night during which they are more awake although we needed to be sure that they stay awake for two hours and it did imply some sensory stimulation. Such control was also very important for two aspects the reviewer did not mention. It allowed us to eliminate from our listing circadian structures activated during the day by the light or structures activated by the sleep deprivation induced. Therefore, we added a group of four Wday-Wnight mice to the study. We now provide analysis of these mice and compare the results obtained between the two conditions as well as with the previous groups of mice. We show that the results obtained with the Wnight condition are for nearly all structures not significantly different that those obtained in the Wday condition indicating that the two conditions are similar and represent a state of natural wake.

*4. Time Window for Neuronal Labeling: The time window for labeling neurons activated during wakefulness should be more clearly defined. While it is suggested that 4-OHT labels neurons activated over a 2 to 4-hour period, confirming the duration of 4-OHT's efficacy is crucial. This will ensure that the method specifically labels neurons activated during wakefulness, rather than those involved in other behaviors (e.g., stress responses) that may occur after drug injection.*

Such a point has been already addressed in the papers describing the method. It has been shown that 4-OHT is binding to CreERT2 expressed at the time of injection. It has also been shown that Tdtomato labeling is induced in a window of less than 6h around the injection. Our work is showing that Tdt and cFos labeling are very well correlated and is therefore clearly showing that the two methods are similar although some differences can be seen.

*5. Clarity of Figures: Certain figures, such as Figure 3, require improvement in clarity. Figure 3 appears blurry, and the layout of the y-axis legend is overcrowded. These issues should be addressed to enhance readability and the overall interpretation of the figures.*

We did review our figures and found that Figure 3 (now 5) was blurry and updated it. The other figures do not seem blurry. However, this is the case when looking at the low quality figures included in the PDF. A link is provided on each of them to get the high quality versions.

*6. Clinical Implications: The authors should consider exploring the potential clinical implications of their findings, particularly in the context of hypersomnia and other sleep disorders. This could significantly enhance the impact of the study. For example, investigating whether treatment strategies can be personalized based on individual neural activation profiles would offer an interesting avenue for future research and clinical application.*

It is an important implication indeed of our study. we started to discuss with clinicians who are treating their patients with the two drugs to see whether they got different feedback from them depending on the treatment. It seems that indeed it might be the case but there is a need for a more controlled study to determine whether indeed the two treatments induce different behaviors and physiological modifications.

Hoping that you will find our corrections satisfactory

Sincerely yours

Claudio Queiroz and Pierre-Hervé Luppi
