# Supplementary material for: Pharmacological and non-pharmacological methods of inducing wakefulness activate distinct neural populations in the mouse brain
Source: PLoS Biol. 2026 Mar 19;24(3):e3003622. doi: 10.1371/journal.pbio.3003622 (PMC13038112; doi:10.1371/journal.pbio.3003622)
Supplement: Attachment — Submitted filename: Responsetoreviewers.docx [file pbio.3003622.s017.docx]

**Pierre-Hervé LUPPI, DR1 - CNRS**

luppi@sommeil.univ-lyon1.fr

+33 (0)4 81 10 65 79

**Equipes de recherche**

**BIORAN**

*Luc Zimmer*

**CAP**

*Nicolas Grimault*

*& Fabien Perrin*

**CMO**

*Nathalie Buonviso*

*& Emmanuelle Courtiol*

**COPHY**

*Mathilde Bonnefond*

*& Jérémie Mattout*

**EDUWEL**

*Jean-Philippe Lachaux*

*& Antoine Lutz*

**ENES**

*Nicolas Mathevon*

**FLUID**

*Jean-François Ghersi-Egea*

**FORGETTING**

*Gaël Malleret*

*& Paul Salin*

**GENDEV**

*Patrick Edery*

*& Sylvie Mazoyer*

**IMPACT**

*Alessandro Farne*

*&Denis Pélisson*

**NEUROPAIN**

*Luis Garcia-Larrea*

*& Roland Peyron*

**NEUROPOP**

*Nathalie Mandairon*

*& Moustafa Bensafi*

**PAM**

*Anne Caclin*

*& Perrine Ruby*

**PSYR2**

*Jérôme Brunelin*

*& Eric Fakra*

**SLEEP**

*Christelle Peyron*

*& Pierre-Hervé Luppi*

**TIGER**

*Laurent Bezin*

*& Sylvain Rheims*

**TRAJECTOIRES**

*Laure Pisella*

*& Yves Rossetti*

**WAKING**

*Jian-Sheng Lin*

*& Laurent Seugnet*

Lyon, March 5, 2026

Dear Editor,

Please find enclosed our revised manuscript named: Pharmacological and non-pharmacological methods of inducing wakefulness activate distinct neural populations in the mouse brain

We detail below our answers to reviewer 4.

Responses to Reviewer 4:

*Reviewer #4: This study systematically compares whole-brain neural activation patterns between drug-induced and natural wakefulness using the TRAP2 transgenic mouse model. The revised manuscript has effectively addressed all previous concerns and presents strong innovation, supported by comprehensive and robust data.*

We thank the reviewer for his very positive feedback.

*Minor revisions are suggested to further enhance its quality, as follows:*

1. *The comparison with Hasan's 2009 study remains somewhat superficial, as it currently focuses solely on differences in dosage and analytical approaches. It is recommended to incorporate direct comparisons of key findings—including co-activated brain regions and the extent of overlap among differentially activated areas—and to more clearly elaborate on the methodological and interpretative advances of the present work relative to this prior study.*

We now include in the Discussion a detailed comparison between our study and that of Hasan et al (Lines 518-542).

*2. The discussion section does not adequately address the unexpected observation of "insufficient activation of the classical arousal system." To deepen the depth and expand the scope of the discussion, it would be valuable to explore potential explanations in the context of existing literature, such as the specificity of drug dosage, variations in animal model characteristics, or distinctions between different subtypes of arousal states.*

We now discuss why the classical wake systems are not strongly activated in our study: In summary, our data indicate that classical wake-promoting systems such as the LC noradrenergic and the VTA dopaminergic neurons are not strongly activated during long-lasting wakefulness induced by modafinil, solriamfetol and sensory stimulation. It might be hypothesized that they are implicated in inducing arousal after acute stimuli and not long-lasting wake although additional experiments are needed to confirm such hypothesis.

In summary, we hope we did address all requests of the reviewers in the new version.

Hoping that you will find our corrections satisfactory

Sincerely yours

Claudio Queiroz and Pierre-Hervé Luppi
